# Relative Contributions of Intracranial Pressure and Intraocular Pressure on Lamina Cribrosa Behavior

**DOI:** 10.1155/2019/3064949

**Published:** 2019-03-17

**Authors:** Junfei Tong, Deepta Ghate, Sachin Kedar, Linxia Gu

**Affiliations:** ^1^Department of Mechanical and Materials Engineering, University of Nebraska-Lincoln, Lincoln, NE 68588-0656, USA; ^2^Stanley Truhlsen Eye Institute, University of Nebraska Medical Center, Omaha, NE 68105-1119, USA; ^3^Department of Neurological Sciences, University of Nebraska Medical Center, Omaha, NE 68198-8440, USA

## Abstract

**Purpose:**

To characterize the relative contributions of intraocular pressure (IOP) and intracranial pressure (ICP) on lamina cribrosa (LC) behavior, specifically LC depth (LCD) and LC peak strain.

**Methods:**

An axially symmetric finite element model of the posterior eye was constructed with an elongated optic nerve and retro-orbital subarachnoid space ensheathed by pia and dura mater. The mechanical environment in LC was evaluated with ICP ranging from 5 to 15 mmHg and IOP from 10 to 45 mmHg. LCD and LC peak strains at various ICP and IOP levels were estimated using full factorial experiments. Multiple linear regression analyses were then applied to estimate LCD and LC peak strain using ICP and IOP as independent variables.

**Results:**

Both increased ICP and decreased IOP led to a smaller LCD and LC peak strain. The regression correlation coefficient for LCD was −1.047 for ICP and 1.049 for IOP, and the ratio of the two regression coefficients was −1.0. The regression correlation coefficient for LC peak strain was −0.025 for ICP and 0.106 for IOP, and the ratio of the two regression coefficients was −0.24. A stiffer sclera increased LCD but decreased LC peak strain; besides, it increased the relative contribution of ICP on the LCD but decreased that on the LC peak strain.

**Conclusions:**

ICP and IOP have opposing effects on LCD and LC peak strain. While their effects on LCD are equivalent, the effect of IOP on LC peak strain is 3 times larger than that of ICP. The influences of these pressure are dependent on sclera material properties, which might explain the pathogenesis of ocular hypertension and normal-tension glaucoma.

## 1. Introduction

Glaucoma is the second leading cause of blindness worldwide. Elevated intraocular pressure (IOP) is the major risk factor for the development of glaucoma [1, 2]. High IOP causes abnormal displacement and strain in the optic nerve head (ONH), specifically within the load-bearing component lamina cribrosa (LC). Excessive displacement and strains in LC have been associated with optic nerve damage including retinal nerve fiber layer thinning [3, 4], axonal damage [5, 6], retinal ganglion cell damage [5, 7], and gene expression alternations in the extracellular matrix [8]. However, 30–60% of glaucoma subjects have normal IOP and are labeled normal-tension glaucoma [9, 10]. This indicates that other factors besides elevated IOP, such as intracranial pressure (ICP), may contribute to the development of glaucoma. In several retrospective studies, glaucoma patients were found to have significantly lower ICP than controls [11, 12]. In experimental studies, artificially lowering ICP in monkeys produced an optic neuropathy that resembled glaucoma [13].

The LC separates IOP compartment from ICP compartment, which creates a pressure differential across the LC called translaminar pressure difference (TLPD) [14]. TLPD, defined as the difference between IOP and ICP, assumes that ICP and IOP produce equal and opposite biomechanical effects on LC [15, 16]. Since the optic nerve subarachnoid space is located along the periphery of LC, the loading effect of ICP on LC is likely to be different from that of IOP. Hence, we hypothesize that ICP and IOP affect LC depth (LCD) and LC peak maximum principal strain (hereafter referred to as LC peak strain) differently. The aim of this work was to estimate and compare the relative contributions of ICP and IOP on LCD and LC peak strain using the finite element method.

## 2. Materials and Methods

### 2.1. Finite Element Model Construction

**Figure 1:**
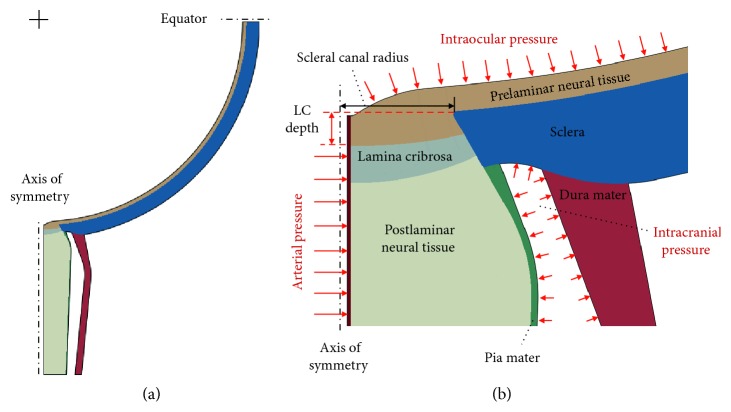
Geometry of the finite element model. (a) Full view. (b) Zoom-in view of the ONH.

An axially symmetric finite element model of the posterior eye (Figure 1) was constructed by modifying established models published in literature [17, 18]. We extended the length of the optic nerve (intraocular and intraorbital segments) to 9 mm, capturing the entire length of the central retinal vessel [19]. The sclera was considered as a 0.78 mm thick spherical shell with an internal radius of 12.22 mm [20]. We attenuated the sclera down to a tapered edge with a thickness of 0.5 mm and a taper angle of 30° from the axis of symmetry, which then formed a scleral canal with a diameter of 1.98 mm at its anterior opening [21]. The perpendicular distance from the scleral canal at its anterior opening to the lowest anterior LC point (283 *μ*m) was defined as LCD (Figure 1(b)). The prelaminar neural tissue, with a thickness of 0.22 mm at the equator, extended till the anterior surface of LC with a contoured shape [17]. The LC was modeled as a spherical sector. The diameter of the anterior LC surface was set as 2.07 mm with thickness at the axis of symmetry as 0.3 mm, producing an anterior surface area of 3.46 mm^2^ [22]. The postlaminar neural tissue below the LC, ensheathed by the pia mater, had a diameter of 3.16 mm and 2.7 mm at 3 mm and 9 mm posterior to the globe, respectively [23]. The thickness of the pia mater decreased from 0.14 mm at the junction of peripapillary sclera to 0.06 mm at 0.5 mm posterior to the globe and remained constant thereafter [24]. The thickness of dura mater decreased from 0.75 mm at the junction of the sclera to 0.4 mm at 3 mm posterior to the globe and remained constant thereafter [25, 26]. The subarachnoid space between pia and dura mater was 0.36 mm at the junction of peripapillary sclera [25], increased to the maximum width of 0.86 mm at 3 mm posterior to the globe, and decreased to 0.52 mm at 9 mm posterior to the globe [23]. The central retinal vessel had an outer diameter of 0.14 mm and a wall thickness of 0.03 mm [27, 28].

**Figure 2:**
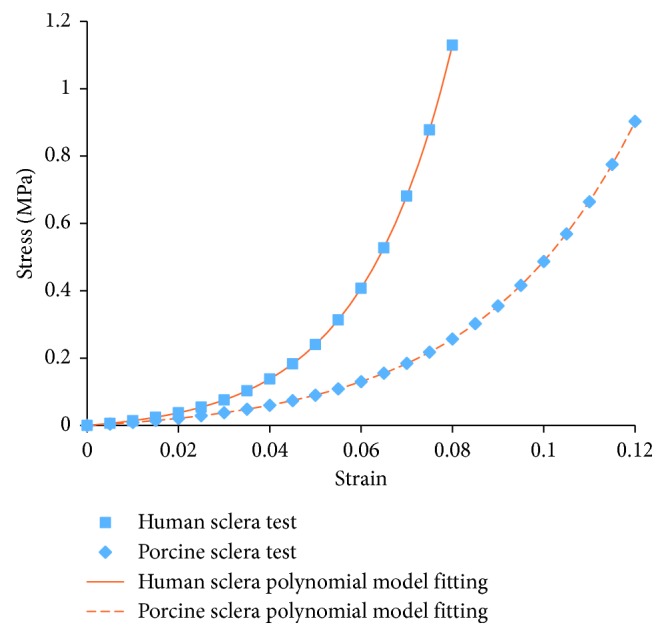
Material properties of sclera.

The material properties of sclera were adopted based on the uniaxial tests by Schultz et al. [29]. A second-order hyperelastic isotropic constitutive model was applied to fit the test data (Figure 2). Its constitutive relation was represented by the following polynomial strain energy density function *U* as

(1) $$\begin{array}{c} U=C_{10}\cdot \left(I_{1}-3\right)+C_{01}\cdot \left(I_{2}-3\right)+C_{11}\cdot \left(I_{1}-3\right)\cdot \left(I_{2}-3\right)+C_{20}\cdot {\left(I_{1}-3\right)}^{2}+C_{02}\cdot {\left(I_{2}-3\right)}^{2}, \end{array}$$

where *C*_*ij*_ are the material coefficients determined from the experimental data, while *I*_1_ and *I*_2_ are the first and second invariants of the Cauchy–Green tensor, respectively. *I*_1_=*λ*_1_^2^+*λ*_2_^2^+*λ*_3_^2^, and *I*_2_=1/*λ*_1_^2^+1/*λ*_2_^2^+1/*λ*_3_^2^. *λ*_*i*_ was the principal stretch ratio. The calculated material constants for the human sclera specimens were *C*_10_=0.582, *C*_01_=−0.381,  *C*_11_=−47069.119, *C*_20_=22867.828, and *C*_02_=24265.006. For porcine sclera specimens, the fitted material constants were *C*_10_=0.893, *C*_01_=−0.755,  *C*_11_=−4622.787, *C*_20_=2223.850, and *C*_02_=2413.072.

All other ONH components were assumed as isotropic, linear-elastic, and incompressible materials with a Poisson's ratio *υ* = 0.49. Young's modulus, as obtained from literature, was 0.03 MPa for both prelaminar and postlaminar neural tissues, 0.3 MPa for LC, 3 MPa for pia mater, and 9 MPa for dura mater [17, 18, 30–32].

We applied an ICP ranging from 5 to 15 mmHg to the subarachnoid space, representing the ICP fluctuation within the normal adults [33]. An IOP ranging from 10 to 45 mmHg, representing the IOP fluctuation within the glaucomatous patients, was applied along the interior surface of the eye, i.e., prelaminar neural tissue [34]. Central retinal arterial pressure was held constant at 55 mmHg [35]. The equator of eyeball in Figure 1(a) was constrained to allow radial movement only, corresponding to anterior-posterior restraint from the extraocular muscles.

The ONH model was meshed using 4-node bilinear axisymmetric quadrilateral shell elements with reduced integration and hourglass control (CAX4R). A mesh convergence test was conducted, and the minimum mesh size of 20 *μ*m was chosen. The baseline model consisted of a total of 88,785 nodes and 87,060 elements. All simulations were carried out using ABAQUS/Standard 6.13 (Simulia, Providence, RI, USA).

### 2.2. Full Factorial Experiment Design

We applied a mixed-level full factorial experiment design using ICP and IOP as independent variables. We considered three levels of ICP (5, 10, and 15 mmHg) and four levels of IOP (5, 15, 25, and 45 mmHg), which resulted in 12 simulation scenarios. Based on the results of these 12 simulation scenarios, multiple linear regression models were applied to quantitatively estimate the relative contributions of ICP and IOP on LCD and LC peak strain. To avoid numerical artifacts, the peak strain was defined as the average of strain values in the 5% of the tissue volume with the highest strain. We calculated the ratio of the two regression coefficients (ICP/IOP) for LCD and LC peak strain to estimate the relative contributions of ICP and IOP for these parameters. A ratio (absolute value) larger than 1 indicates that the contribution of ICP is more than that of IOP.

### 2.3. Influence of ICP and IOP Fluctuations

From the 12 simulation scenarios, we used 3 scenarios to assess the effects of ICP increase or IOP decrease on LC anterior surface displacement and LC peak strain. The LC anterior surface displacements were calculated at LC center and LC quarter-point referenced to the anterior insertion point of LC. A positive value indicates anterior shift while a negative value indicates a posterior shift. The baseline model was designated as ICP at 5 mmHg and IOP at 15 mmHg. We determined LCD and LC peak strain changes by inducing either 5 mmHg ICP elevation (ICP = 10 mmHg, IOP = 15 mmHg) or 5 mmHg IOP reduction (ICP = 5 mmHg, IOP = 10 mmHg).

### 2.4. Influence of Sclera Material Property

The influence of sclera material property on ONH biomechanics was evaluated by changing the aforementioned human sclera material properties to the porcine sclera material properties in the full factorial experiments.

## 3. Results

### 3.1. Full Factorial Computational Experiments

The LCD and LC peak strains obtained from the 12 simulation scenarios are listed in Tables 1 and 2. The regression equations modeled for LCD and LC peak strain using data listed below show an excellent fitting (*R* ^2^∼1):

(2) $$\begin{array}{c} \text{LCD}\left(µ\text{m}\right)=280.9-1.047\;*\text{ ICP}+1.049\;*\text{ IOP},\;\left(R^{2}=0.9997\right),\\ \text{LC peak strain}\left(\%\right)=0.775-0.025\;*\text{ ICP}+0.106\;*\text{ IOP},\;\left(R^{2}=0.9994\right). \end{array}$$

### 3.2. Influence of ICP and IOP Fluctuations

**Figure 3:**
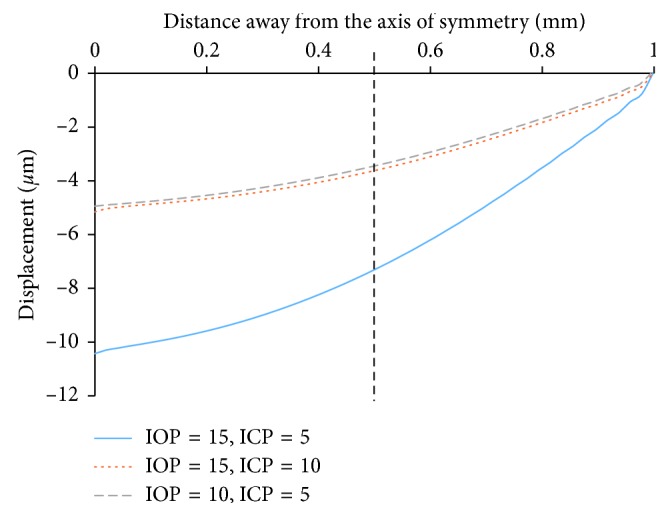
LC anterior surface displacement (dashed black line at one-half of the LC radius indicates the location of LC quarter-point). ^*∗*^Units for IOP and ICP are mmHg.

Compared to the baseline model, the model with 5 mmHg ICP elevation and the model with 5 mmHg IOP reduction induced a similar anterior LC shift as shown in Figure 3. Specifically, 5 mmHg ICP elevation resulted in an anterior shift of 5.2 *μ*m at the center and 3.6 *μ*m at the quarter-point location. A 5 mmHg IOP reduction led to an anterior shift of 5.5 *μ*m at the center and 3.7 *μ*m at the quarter-point location. The LC shift in the quarter-point compared to the LC shift in the center was 69.2% in the elevated ICP model and 67.3% in the decreased IOP model, respectively.

**Figure 4:**
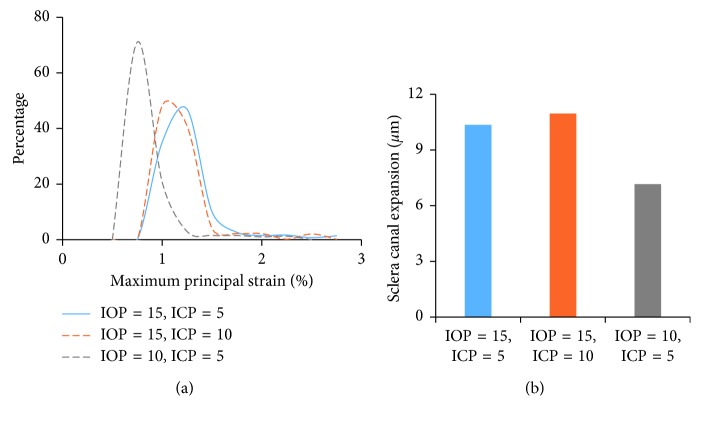
(a) Probability distribution of the maximum principal strain in LC. (b) Scleral canal expansion. ^*∗*^Units for IOP and ICP are mmHg.

The maximum principal strain probability distributions of LC in the baseline model, the model with 5 mmHg ICP elevation, and the model with 5 mmHg IOP reduction were depicted in Figure 4(a). All distributions had a positive skew with a tail extending to the right, and IOP reduction was found to decrease the strain in LC dramatically compared to ICP elevation.  Figure 4(b) shows the scleral canal expansion in three loading cases. The baseline model induced a scleral canal expansion of 10.4 *μ*m, compared with 11.0 *μ*m in the model with elevated ICP and 7.2 *μ*m in the model with decreased IOP, respectively.

### 3.3. Effect of Scleral Property

The results of LCD and LC peak strain for the 12 simulation cases using porcine sclera material property are listed in Tables 3 and 4. The regression equations modeled for LCD and LC peak strain also show an excellent fitting (*R* ^2^∼1):

(3) $$\begin{array}{c} \text{LCD}\left(µ\text{m}\right)=279.7-1.045\;*\text{ ICP}+0.902\;*\text{ IOP},\;\left(R^{2}=0.9986\right),\\ \text{LC peak strain}\left(\%\right)=0.818-0.021\;*\text{ ICP }+0.124\;*\text{ IOP},\;\left(R^{2}=0.9996\right). \end{array}$$

Compared to models using human sclera material property, models using the softer porcine sclera showed a 2–8 *μ*m reduced LCD and 15–19% higher LC peak strain. The ratio of the two regression coefficients for LCD and LC strain is summarized in Table 5. The softer porcine sclera led to an increased influence of ICP on LCD but a reduced influence of ICP on LC peak strain.

## 4. Discussion

The results from our experimental work show that both IOP and ICP significantly influence the behavior of LC in different ways. We used LCD as a surrogate for the clinical finding of optic disc cupping in glaucoma [3, 36] and LC peak strain due to its association with glaucoma pathogenesis [8, 37].

Our models show that in humans, IOP and ICP have an equal but opposite effect on LCD which suggests that LCD is determined by TLPD. This is consistent with the observations in human subjects undergoing medically necessary lumbar puncture, where it was shown that anterior LCD was correlated with the TLPD, but neither ICP nor IOP alone [15]. On the other hand, we found that the effect of ICP on LC peak strain was 24% compared to the effect of IOP. To verify our results, we calculated LC peak strain by applying our regression model to experimentally measured IOP and ICP datasets from prior publications [11, 12]. We found that the glaucomatous group had a significantly higher strain than the control group, which validates our model. A detailed description of the data processing is available in the Supplementary Material.

We found that a 5 mmHg ICP elevation or 5 mmHg IOP reduction resulted in a similar anterior shift of LC. However, a 5 mmHg ICP elevation had much less influence on LC strain than IOP reduction (5% decrease vs 24% decrease). We also found that the quarter-point LC displacement was 2/3^rd^ of central LC displacement for both ICP elevation and IOP reduction (69.2% and 67.3%, respectively), which was consistent with the experimental study by Yan et al. where the value was between 66% and 69% [38].

Since the scleral in-wall hoop stress is believed to influence LC biomechanics, we studied the scleral canal expansion induced by the changes in IOP and ICP [5, 39]. We found that decreased IOP reduces the scleral canal expansion while elevated ICP increases the scleral canal expansion, which explains the different contributions of ICP and IOP on LC peak strain. Decreased IOP causes anterior LC shift and scleral canal narrowing, both resulting in a reduced LC strain. Increased ICP causes anterior LC shift but increased scleral canal expansion. While the former leads to a decreased LC strain, the latter causes an increased LC strain. These two opposite effects diminish the role of ICP on the LC strain, thus resulting in a much smaller contribution of ICP on LC strain compared to IOP.

We also found that sclera material property significantly influences LCD and LC peak strain, consistent with recent reports that scleral material property is an influential factor in ONH biomechanics [40]. Aging results in an increased scleral stiffness; for example, the stiffness of sclera at the age of 80 is three times larger than that at the age of 40 [41]. We found that for a given ICP and IOP, a stiffer sclera resulted in a larger LCD and a smaller LC peak strain. This is consistent with previous studies that stiffer sclera (18.4 MPa) induced 4 *μ*m greater LCD compared to softer sclera (5.3 MPa) [42] and LC peak strain in the elderly decreased by 0.048%∼0.062% each year [37].

We also found that scleral material property influences the relative contributions of ICP and IOP on LCD and LC peak strain. In human sclera, ICP and IOP have equivalent effect on LCD, while in softer porcine sclera, the ICP contributes 16% more than IOP. On the contrary, the absolute ratio of the two regression coefficients (ICP/IOP) on LC peak strain decreases from 0.24 in human sclera to 0.17 in softer porcine sclera. This suggests that as the human sclera stiffens with age, the influence of IOP on LC strain decreases compared to ICP. Considering ICP was also found to decrease with age, this finding may explain the increase of normal-tension glaucoma incidence with age, such as from 0.2% in 43∼54 years age group 1 to 1.6% in group older than 75 years [9, 43]. Our findings also suggest that current glaucoma treatment strategies focused on IOP reduction to delay ganglion cell loss and progression of glaucoma, presumably by reducing LC strain [44].

The unequal contribution of IOP and ICP on LC peak strain can explain the increased incidence of glaucomatous visual field defects seen in high-resistance wind instrument players [45]. The playing of high-resistance wind instrument is similar to Valsalva maneuver. Zhang et al. showed elevation of both IOP and ICP during the Valsalva maneuver; however, ICP elevation is significantly higher compared to IOP (10.5 ± 2.7 mmHg vs. 1.9 ± 2.4 mmHg) [46]. We propose that players who develop optic nerve damage likely do so because the LC strain increase caused by IOP elevation is not compensated by the higher elevated ICP. Further, players at a younger age with softer sclera may be at a higher risk for glaucomatous damage due to a higher impact of IOP compared to ICP.

Glaucomatous optic neuropathy is characterized by pathologic cupping, indicating LC remodeling and progressive loss of retinal ganglion cell axon loss, which is commonly seen in patients with elevated IOP [36]. However, increased optic disc cupping may also be seen in other types of optic neuropathies such as normal-tension glaucoma and certain patients with other forms of optic neuropathy in the absence of elevated IOP [47, 48]. Based on the result in this work that ICP and IOP contributes equally on the LCD, those optic disc cupping without elevated IOP may be attributed to a decreased ICP.

Our study has several limitations. First, we used a simplified geometric finite element model with tissue dimensions adopted from various studies. Thus, our regression models should be treated as generic rather than individual-specific. We did not consider the influence of ONH geometrical parameters such as radius of the eye and thickness of sclera shell which have been shown to influence ONH biomechanics [49]. On the contrary, based on Hua's work, the other geometrical parameters' influence on the LC strain is relatively negligible compared to that of sclera modulus; thus, only the sclera modulus influence was investigated in this work [40]. It was believed that our conclusion would be still valid when a varied geometry of ONH was considered.

Second, we assumed linear-elastic and isotropic tissue material property to simplify our model. Studies have shown that collagenous tissues such as LC, sclera, and dura mater exhibit nonlinear and anisotropic behavior [50–52]. In our future work, we plan to build more comprehensive models incorporating material properties with nonlinear and anisotropic behavior.

Lastly, we studied the influence of acute changes in ICP and IOP. Our results cannot be extrapolated to subjects with chronic ICP changes or IOP changes, which presumably results in tissue remodeling [36, 53].

## 5. Conclusion

We found that both ICP and IOP influence LC morphology and biomechanics. While ICP and IOP have an equivalent effect on LCD, the effect of IOP on LC peak strain is 3 times larger than that of ICP. The influences of these pressure are dependent on sclera material properties, which might explain the pathogenesis of ocular hypertension and normal-tension glaucoma.

## Figures and Tables

**Table 1:** LCD in the trial runs of full factorial simulation experiment with human sclera material property.

LCD (*μ*m)		IOP (mmHg)			
		10	15	25	45
ICP (mmHg)	5	286.49	291.38	301.46	322.88
	10	281.24	286.19	296.33	317.86
	15	275.82	280.78	291.04	312.69

**Table 2:** LC peak strain in the trial runs of full factorial simulation experiment with human sclera material property.

LC peak strain (%)		IOP (mmHg)			
		10	15	25	45
ICP (mmHg)	5	1.67	2.21	3.29	5.46
	10	1.58	2.10	3.14	5.27
	15	1.52	2.00	3.01	5.10

**Table 3:** LCD in the trial runs of full factorial simulation experiment with porcine sclera material property.

LCD (*μ*m)		IOP (mmHg)			
		10	15	25	45
ICP (mmHg)	5	284.13	287.91	296.27	315.29
	10	278.92	282.78	291.21	310.23
	15	273.46	277.39	285.90	305.06

**Table 4:** LC peak strain in the trial runs of full factorial simulation experiment with porcine sclera material property.

LC peak strain (%)		IOP (mmHg)			
		10	15	25	45
ICP (mmHg)	5	1.92	2.55	3.81	6.33
	10	1.83	2.45	3.69	6.17
	15	1.79	2.38	3.57	6.02

**Table 5:** Ratio of the two regression coefficients (ICP/IOP) in absolute value.

	Human sclera	Porcine sclera
LCD	1.00	1.16
LC peak strain	0.24	0.17

## Data Availability

The data used to support the findings of this study are available from the corresponding author upon request.

## Supplementary Materials

Supplementary Materials Table 1: ICP and IOP measured by Berdahl et al. Table 2: pressure datasets from Ren's publication. Figure 1: secondary analysis using measured ICP and IOP from experiment by Berdahl et al. Figure 2: secondary analysis using measured ICP and IOP from experiment by Ren et al.

## References

[1] Gordon M. O. (2002). The ocular hypertension treatment study: baseline factors that predict the onset of primary open-angle glaucoma. *Archives of ophthalmology*.

[2] Sommer A., Tielsch J. M., Katz J. (1991). Relationship between intraocular pressure and primary open angle glaucoma among white and black Americans: the Baltimore Eye Survey. *Archives of ophthalmology*.

[3] Furlanetto R. L., Park S. C., Damle U. J. (2013). Posterior displacement of the lamina cribrosa in glaucoma: in vivo interindividual and intereye comparisons. *Investigative Opthalmology & Visual Science*.

[4] Lee E. J., Kim T.-W., Kim M., Kim H. (2015). Influence of lamina cribrosa thickness and depth on the rate of progressive retinal nerve fiber layer thinning. *Ophthalmology*.

[5] Burgoyne C. F., Crawford Downs J., Bellezza A. J., Francis Suh J.-K., Hart R. T. (2005). The optic nerve head as a biomechanical structure: a new paradigm for understanding the role of IOP-related stress and strain in the pathophysiology of glaucomatous optic nerve head damage. *Progress in Retinal and Eye Research*.

[6] Quigley H. A. (1981). Optic nerve damage in human glaucoma: II. The site of injury and susceptibility to damage. *Archives of ophthalmology*.

[7] Anderson D. R., Hendrickson A. (1974). Effect of intraocular pressure on rapid axoplasmic transport in monkey optic nerve. *Investigative Ophthalmology & Visual Science*.

[8] Kirwan R. P., Fenerty C. H, Crean J., Wordinger R. J., Clark A. F, O’Brien C. J. (2005). Influence of cyclical mechanical strain on extracellular matrix gene expression in human lamina cribrosa cells in vitro. *Molecular Vission*.

[9] Klein B. E. K., Klein R., Sponsel W. E. (1992). Prevalence of glaucoma: the Beaver Dam Eye Study. *Ophthalmology*.

[10] Shiose Y., Kitazawa Y., Tsukahara S. (1991). Epidemiology of glaucoma in Japan—a nationwide glaucoma survey. *Japanese Journal of Ophthalmology*.

[11] Berdahl J. P., Allingham R. R., Johnson D. H. (2008). Cerebrospinal fluid pressure is decreased in primary open-angle glaucoma. *Ophthalmology*.

[12] Ren R., Jonas J. B., Tian G. (2010). Cerebrospinal fluid pressure in glaucoma: a prospective study. *Ophthalmology*.

[13] Yang D., Fu J., Hou R. (2014). Optic neuropathy induced by experimentally reduced cerebrospinal fluid pressure in monkeys. *Investigative Opthalmology & Visual Science*.

[14] Morgan W. H., Chauhan B. C., Yu D. Y., Cringle S. J., Alder V. A., House P. H. (2002). Optic disc movement with variations in intraocular and cerebrospinal fluid pressure. *Investigative ophthalmology & visual science*.

[15] Lee D. S., Lee E. J., Kim T.-W. (2015). Influence of translaminar pressure dynamics on the position of the anterior lamina cribrosa surface. *Investigative Opthalmology & Visual Science*.

[16] Hayreh S. S. (2009). *Cerebrospinal Fluid Pressure and Glaucomatous Optic Disc Cupping*.

[17] Sigal I. A., Flanagan J. G., Tertinegg I., Ethier C. R. (2004). Finite element modeling of optic nerve head biomechanics. *Investigative Opthalmology & Visual Science*.

[18] Hua Y., Tong J., Ghate D., Kedar S., Gu L. (2017). Intracranial pressure influences the behavior of the optic nerve head. *Journal of Biomechanical Engineering*.

[19] Singh S., Dass R. (1960). The central artery of the retina I. Origin and course. *British Journal of Ophthalmology*.

[20] Olsen T. W., Aaberg S. Y., Geroski D. H., Edelhauser H. F. (1998). Human sclera: thickness and surface area. *American Journal of Ophthalmology*.

[21] Norman R. E., Flanagan J. G., Rausch S. M. K. (2010). Dimensions of the human sclera: thickness measurement and regional changes with axial length. *Experimental eye research*.

[22] Jonas J., Mardin C. Y., Schlötzer-Schrehardt U., Naumann G. O. (1991). Morphometry of the human lamina cribrosa surface. *Investigative Ophthalmology & Visual Science*.

[23] Xie X., Zhang X., Fu J. (2013). Noninvasive intracranial pressure estimation by orbital subarachnoid space measurement: the Beijing Intracranial and Intraocular Pressure (iCOP) study. *Critical Care*.

[24] Balaratnasingam C., Morgan W. H., Johnstone V., Pandav S. S., Cringle S. J., Yu D.-Y. (2009). Histomorphometric measurements in human and dog optic nerve and an estimation of optic nerve pressure gradients in human. *Experimental Eye Research*.

[25] Jonas J. B., Jonas S. B., Jonas R. A., Holbach L., Panda-Jonas S. (2011). Histology of the parapapillary region in high myopia. *American Journal of Ophthalmology*.

[26] Lang J. (2012). *Clinical Anatomy of the Head: Neurocranium·Orbit·Craniocervical Regions*.

[27] Muraoka Y., Tsujikawa A., Kumagai K. (2013). Age- and hypertension-dependent changes in retinal vessel diameter and wall thickness: an optical coherence tomography study. *American Journal of Ophthalmology*.

[28] Rim T., Choi Y. S., Kim S. S. (2015). Retinal vessel structure measurement using spectral-domain optical coherence tomography. *Eye*.

[29] Schultz D. S., Lotz J. C., Lee S. M., Trinidad M. L., Stewart J. M. (2008). Structural factors that mediate scleral stiffness. *Investigative Opthalmology & Visual Science*.

[30] Jones I. L., Warner M., Stevens J. D. (1992). Mathematical modelling of the elastic properties of retina: a determination of Young’s modulus. *Eye*.

[31] Ozawa H., Matsumoto T., Ohashi T., Sato M., Kokubun S. (2004). Mechanical properties and function of the spinal pia mater. *Journal of Neurosurgery: Spine*.

[32] Bellezza A. (2002). *Biomechanical Properties of the Normal and Early Glaucomatous Optic Nerve Head: An Experimental and Computational Study Using the Monkey Model*.

[33] Smith M. (2008). Monitoring intracranial pressure in traumatic brain injury. *Anesthesia & Analgesia*.

[34] Medeiros F. A., Meira-Freitas D., Lisboa R., Kuang T.-M., Zangwill L. M., Weinreb R. N. (2013). Corneal hysteresis as a risk factor for glaucoma progression: a prospective longitudinal study. *Ophthalmology*.

[35] Lam C. S. P., Donal E., Kraigher-Krainer E., Vasan R. S. (2011). Epidemiology and clinical course of heart failure with preserved ejection fraction. *European Journal of Heart Failure*.

[36] Downs J. C., Roberts M. D., Sigal I. A. (2011). Glaucomatous cupping of the lamina cribrosa: a review of the evidence for active progressive remodeling as a mechanism. *Experimental Eye Research*.

[37] Midgett D. E., Pease M. E., Jefferys J. L. (2017). The pressure-induced deformation response of the human lamina cribrosa: analysis of regional variations. *Acta Biomaterialia*.

[38] Yan D. B., Coloma F. M., Metheetrairut A., Trope G. E., Heathcote J. G., Ethier C. R. (1994). Deformation of the lamina cribrosa by elevated intraocular pressure. *British Journal of Ophthalmology*.

[39] Downs J. C., Roberts M. D., Burgoyne C. F. (2008). The mechanical environment of the optic nerve head in glaucoma. *Optometry and Vision Science*.

[40] Hua Y., Voorhees A. P., Sigal I. A. (2018). Cerebrospinal fluid pressure: revisiting factors influencing optic nerve head biomechanics. *Investigative Opthalmology & Visual Science*.

[41] Coudrillier B., Tian J., Alexander S., Myers K. M., Quigley H. A., Nguyen T. D. (2012). Biomechanics of the human posterior sclera: age-and glaucoma-related changes measured using inflation testing. *Investigative Opthalmology & Visual Science*.

[42] Sigal I. A., Yang H., Roberts M. D., Burgoyne C. F., Downs J. C. (2011). IOP-induced lamina cribrosa displacement and scleral canal expansion: an analysis of factor interactions using parameterized eye-specific models. *Investigative Opthalmology & Visual Science*.

[43] Pedersen S. H., Lilja-Cyron A., Andresen M., Juhler M. (2017). The relationship between intracranial pressure and age—chasing age-related reference values. *World Neurosurgery*.

[44] Heijl A. (2002). Reduction of intraocular pressure and glaucoma progression: results from the Early Manifest Glaucoma Trial. *Archives of Ophthalmology*.

[45] Schuman J. S., Massicotte E. C., Connolly S., Hertzmark E., Mukherji B., Kunen M. Z. (2000). Increased intraocular pressure and visual field defects in high resistance wind instrument players. *Ophthalmology*.

[46] Zhang Z., Wang X., Jonas J. B. (2014). Valsalva manoeuver, intra-ocular pressure, cerebrospinal fluid pressure, optic disc topography: Beijing intracranial and intra-ocular pressure study. *Acta Ophthalmologica*.

[47] Ambati B. K., Rizzo J. F. (2001). Nonglaucomatous cupping of the optic disc. *International Ophthalmology Clinics*.

[48] Gupta P. K., Asrani S., Freedman S. F., El-Dairi M., Bhatti M. T. (2011). Differentiating glaucomatous from non-glaucomatous optic nerve cupping by optical coherence tomography. *Open Neurology Journal*.

[49] Sigal I. A., Flanagan J. G., Ethier C. R. (2005). Factors influencing optic nerve head biomechanics. *Investigative Opthalmology & Visual Science*.

[50] Zhang L., Albon J., Jones H. (2015). Collagen microstructural factors influencing optic nerve head biomechanics. *Investigative Opthalmology & Visual Science*.

[51] Girard M. J. A., Dahlmann-Noor A., Rayapureddi S. (2011). Quantitative mapping of scleral fiber orientation in normal rat eyes. *Investigative Opthalmology & Visual Science*.

[52] Runza M., Pietrabissa R., Mantero S., Albani A., Quaglini V., Contro R. (1999). Lumbar dura mater biomechanics: experimental characterization and scanning electron microscopy observations. *Anesthesia & Analgesia*.

[53] Girard M. J. A., Suh J.-K. F., Bottlang M., Burgoyne C. F., Downs J. C. (2011). Biomechanical changes in the sclera of monkey eyes exposed to chronic IOP elevations. *Investigative Opthalmology & Visual Science*.

