# Supplementary material for: Relative Contributions of Intracranial Pressure and Intraocular Pressure on Lamina Cribrosa Behavior
Source: J Ophthalmol. 2019 Mar 17;2019:3064949. doi: 10.1155/2019/3064949 (PMC6441528; doi:10.1155/2019/3064949)
Supplement: Supplementary Materials — Table 1: ICP and IOP measured by Berdahl et al. Table 2: pressure datasets from Ren's publication. Figure 1: secondary analysis using measured ICP and IOP from experiment by Berdahl et al. Figure 2: secondary analysis using measured ICP and IOP from experiment by Ren et al. [file 3064949.f1.docx]

Our results were used to further interpret the measured ICP and IOP datasets for both the primary open-angle glaucoma group (POAG) and nonglaucomatous controls by Berdahl et. al. [1]. The pressure datasets were summarized in Table 1, and a significantly lower ICP existed in POAG patients compared to controls. The maximum IOP within 24 hours was clearly elevated in POAG patients. The difference between left and right eyes was minimal.

| Table 1. ICP and IOP measured by Berdahl et. al | | | |
| --- | --- | --- | --- |
|  | | Controls | POAG |
| ICP (mmHg) | | 13.0 ± 4.2 | 9.2 ± 2.9 |
| IOP near time of lumbar puncture (LP) (mmHg) | Right eye | 14.9 ± 3.0 | 15.1 ± 4.5 |
|  | Left eye | 14.9 ± 3.3 | 15.5 ± 6.0 |
| Maximum IOP (mmHg) | Right eye | 16.4 ± 2.8 | 24.3 ± 6.1 |
|  | Left eye | 16.6 ± 3.3 | 25.0 ± 7.9 |

The measured IOP and ICP in Table 1. were used to calculate the peak strain in LC using our regression equation 3. The results were then depicted in Fig. 1. It was observed that a lower ICP and slightly higher IOP near the time of LP resulted in a higher LC strain level in POAG patients. As expected, the greatly increased maximum IOP with the same lower ICP led to a significantly higher LC peak strain in POAG patients (P<0.001). Our results quantitatively clarified the hypothesis that strain in LC was biologically significant in the development of glaucoma [2]. One implication is that the IOP should be recorded through a period of at least 24 hours in the in vivo models.


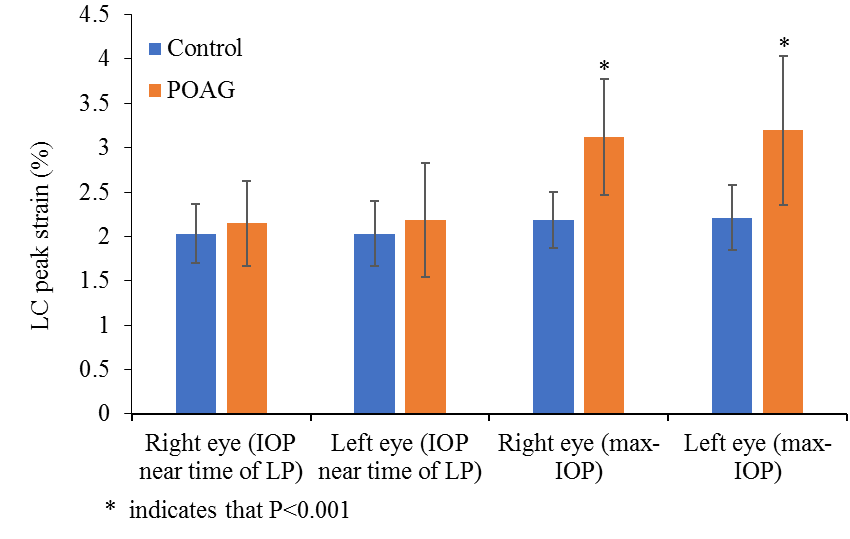


Fig.1 Secondary analysis using measured ICP and IOP from experiment by Berdahl et al.

Similarly, our results were used to further interpret the measured ICP and IOP datasets for normal tension glaucoma (NTG), high tension glaucoma (HTG) and controls by Ren et.al. [3]. The pressure datasets were summarized in Table 2. The IOP measured near the time of LP had fewer alternations in the control group, compared with the maximum IOP recorded within a 24-hour period. A lower ICP and elevated IOP were observed in both NTG and HTG groups compared to controls. In addition, both ICP and IOP were lower in NTG than that in HTG. Both measurements of IOP, i.e., the IOP near the time of LP and the maximum IOP, were used to estimate the peak strain in LC using our regression equation 3. The results were shown in Fig. 2.

| Table 2. Pressure datasets from Ren’s publication | | | |
| --- | --- | --- | --- |
|  | Controls | NTG | HTG |
| ICP (mmHg) | 12.9 ± 1.9 | 9.5 ± 2.2 | 11.7 ± 2.7 |
| IOP near time of LP (mmHg) | 14.3 ± 2.6 | 16.1 ± 1.9 | 24.3 ± 3.2 |
| Maximum IOP(mmHg) | 15.9 ± 2.5 | 18.5 ± 0.9 | 28.5 ± 3.8 |


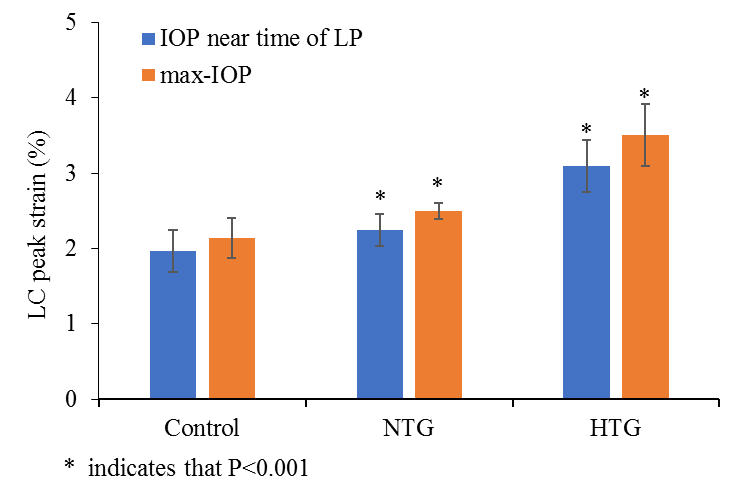


Fig.2 Secondary analysis using measured ICP and IOP from experiment by Ren et al.

Similarly, the peak strain in LC for both NTG and HTG groups were significantly larger than that of controls (P <0.001), which was valid for both IOP measures.

References

1. Berdahl, J.P., R.R. Allingham, and D.H. Johnson, *Cerebrospinal fluid pressure is decreased in primary open-angle glaucoma.* Ophthalmology, 2008. **115**(5): p. 763-768.

2. Sigal, I.A., et al., *Finite element modeling of optic nerve head biomechanics.* Investigative ophthalmology & visual science, 2004. **45**(12): p. 4378-4387.

3. Ren, R., et al., *Cerebrospinal fluid pressure in glaucoma: a prospective study.* Ophthalmology, 2010. **117**(2): p. 259-266.
